# Poly(Lactic Acid) Nanoparticles Targeting α5β1 Integrin as Vaccine Delivery Vehicle, a Prospective Study

**DOI:** 10.1371/journal.pone.0167663

**Published:** 2016-12-14

**Authors:** Bastien Dalzon, Célia Lebas, Gina Jimenez, Alice Gutjahr, Céline Terrat, Jean-Yves Exposito, Bernard Verrier, Claire Lethias

**Affiliations:** 1 Institut de Biologie et Chimie des Protéines, Fédération de Recherche, SFR BioSciences (Unité Mixte de Service 3444/US8) Gerland-Lyon Sud, Université de Lyon 1, Lyon, France; 2 Laboratoire de Biologie Tissulaire et d'Ingénierie Thérapeutique, Centre National de la Recherche Scientifique, Unité Mixte de Recherche, 7 passage du Vercors, Lyon, France; 3 Institut NeuroMyogène, CNRS UMR-5310 INSERM-U1217, Université de Lyon 1, Lyon, France; Helsingin Yliopisto, FINLAND

## Abstract

Biodegradable polymeric nanoparticles are vehicles of choice for drug delivery and have the ability to encapsulate and present at their surface different molecules of interest. Among these bio-nanocarriers, poly(lactic acid) (PLA) nanoparticles have been used as adjuvant and vehicle for enhanced vaccine efficacy. In order to develop an approach to efficient vaccine delivery, we developed nanoparticles to target α5β1 positive cells. We first overproduced, in bacteria, human fibronectin FNIII9/10 recombinant proteins possessing an integrin α5β1 binding site, the RGDS sequence, or a mutated form of this site. After having confirmed the integrin binding properties of these recombinant proteins in cell culture assays, we were able to formulate PLA nanoparticles with these FNIII9/10 proteins at their surface. We then confirmed, by fluorescence and confocal microscopy, an enhanced cellular uptake by α5β1^+^ cells of RGDS-FNIII9/10 coated PLA nanoparticles, in comparison to KGES-FNIII9/10 coated or non-coated controls. As a first vaccination approach, we prepared PLA nanoparticles co-coated with p24 (an HIV antigen), and RGDS- or KGES-FNIII9/10 proteins, followed by subcutaneous vaccine administration, in mice. Although we did not detect improvements in the apparent humoral response to p24 antigen in the serum of RGDS/p24 nanoparticle-treated mice, the presence of the FNIII proteins increased significantly the avidity index of anti-p24 antibodies compared to p24-nanoparticle-injected control mice. Future developments of this innovative targeted vaccine are discussed.

## Introduction

During recent decades, attempts to develop cheap, efficient, easy-to-use, and non-toxic vaccines with less side effects have included the use of new adjuvants, new supporting materials, and new targeting strategies [1]. Among the carriers developed, biodegradable and biocompatible poly(lactic acid) (PLA) nanoparticles have been used to support and to enhance the potential of antigens. Hence, this Food and Drug Administration (FDA) approved biomaterial has been shown to act as a perfect vehicle to carry antigens and to play a safe and non-toxic adjuvant function, either alone or with the loading of pattern recognition receptor (PRR) ligands to increase its potency [2–6].

One of the scientific approaches to efficiently target specific cells is to build a nanomaterial harboring cell-specific ligands on its surface. This is one of the strategies that pathogens use to infect host cells to target available receptors via their external binding ligands. Arg-Gly-Asp (RGD) containing ligands have been used by a large number of viruses [7], this tripeptide motif being the ligand of various integrins associated with membrane rafts that are sites of cellular entry for these pathogens [8]. RGD peptides are also used for the diagnosis and development of cancer therapy projects [9]. Hence, the tripeptide RGD is one of the most useful ligands to target cells presenting at their surface RGD-binding integrins such as α3β1, α5β1, αVβ1, αVβ3, αVβ5, αVβ6, αVβ8, αIIβ3, αMβ2,and αLβ2, and is widely used in drug delivery therapy [10–11].

Among the known integrin-ligand interactions, the fibronectin and its interaction with α5β1 integrin, via an RGDS sequence has been the subject of numerous studies [12–13]. The RGDS sequence is located in the C-terminal region of FNIII domain 10 (FNIII10), and its interaction with RGD-binding integrins is enhanced by the synergy site Pro-His-Ser-Arg-Asn (PHSRN), located in the FNIII-9 domain [13]. This integrin-fibronectin interaction plays important roles during development, as, for example, during cardiovascular development [14–15]. In adults, the expression of these proteins is less pronounced. The α5β1 integrin is present in microfold (M) cells of the digestive track, in dermal dendritic cells [16], and more generally is present in a wide range of tissues as a cell receptor for cellular (extracellular matrix) fibronectin. Its overexpression has also been identified in numerous tumors, or during tissue regeneration, such as skin healing [17–18]. Because of these properties, fibronectin or recombinant fragments of this protein have already been used in the design of biomaterials [19–20].

To take advantage of the adjuvant function and carrier capacity of PLA nanoparticles, we designed these vehicles to target α5β1 positive cells. For this, we overproduced, in bacteria, the FNIII9/10 domains of human fibronectin in its wild-type form, including the RGDS sequence, or in a mutated form with the fibronectin-ligand sequence replaced by KGES, which prevents its binding to α5β1 integrin receptors. After coating the nanoparticle surface with these recombinant proteins, we showed that RGDS-PLA nanoparticles were more efficiently taken up by cells harboring α5β1 integrin receptors on their cell surface than uncoated or mutated KGES-coated nanoparticles. As a first approach, a subcutaneous vaccine model was developed to take advantage of the presence of dendritic cells presenting α5β1 integrin receptors on their surface in skin and to a less-extent in subcutaneous tissues, and their potential to migrate to peripheral draining lymph nodes [21–22]. Mice were injected with different types of nanoparticles, coated with p24, an HIV antigen, and with or without RGDS- or KGES-FNIII9/10 recombinant proteins, and the antigenic potential of these formulations was estimated.

## Materials and Methods

### Cell lines and reagents

The human fibrosarcoma cell line HT-1080 (ATCC^®^ CCL-121^™)^ was maintained as recommended by the ATCC. The integrin blocking antibodies were purchased from Millipore (Molsheim, France) and included a monoclonal anti-α (MAB1956, 10 μg/mL), an anti-β1 (MAB1959, 10 μg/mL), and an anti-α2 (MAB1950, 10 μg/mL). HIV-1 p24 protein was produced and purified by PX’Therapeutics (Protein’eXpert, Grenoble, France).

### Recombinant proteins

DNA matrices used to generate, by PCR, the sequence encoding FNIII domains 9 and 10 of human fibronectin were a gift from Dr Helen J Mardon ([12]; nucleotides 4599–5162, NCBI reference sequence NM_212482.2). Oligonucleotide sequences were: 5’-TAT**GAATTC**AGAAAACAGGTCTTGATTCCCCAAC-3’ (forward), and 5’-TAT**CTGCAG**TTCAGTTCGGTAATTAATGG-3' (reverse), where the *Eco* RI and *Pst* I restriction endonuclease sites used for cloning are in bold. PCR was carried out with the proofreading *pfu* DNA polymerase, and the *Eco* RI-*Pst* I fragments were cloned into a pT7/7 derivative that allows the production in bacteria of recombinant proteins with a 6-His tag at their C-terminus. Recombinant protein was expressed in the BL21(DE3) *Escherichia coli* strain, and soluble peptides were purified, as previously described [23], except that the final dialysis was done against 20 mM phosphate buffer pH7. Endotoxins were removed from purified proteins using an affinity column (PierceTM high capacity endotoxin removal resin; Thermo Fisher Scientific, Villebon sur Yvette, France) following the manufacturer's instructions. Proteins were next dialyzed against PBS (pH 7.4), and the endotoxin level was determined using Endosafe**®**-PTSTM (Charles River, Ecully, France), which consists of a spectrophotometer and a FDA licensed reagent cartridge based on the limulus amebocyte lysate (LAL).

### PLA nanoparticles

PLA, BODIPY® 500/510 C_4_, C_9_ (0.2%, ThermoFisher Scientific, Villebon sur Yvette, France) (5-Butyl-4,4-Difluoro-4-Bora-3a,4a-Diaza-*s*-Indacene-3-Nonanoic Acid) and Coumarin 6 (0.2%; Sigma, Saint-Quentin-Fallavier, France) PLA nanoparticle batches were provided by Adjuvatis (Lyon, France). Blank and fluorescent modified PLA nanoparticles were made by nanoprecipitation, as previously described [24–26]. PLA nanoparticle batches were characterized for their hydrodynamic diameter and their zeta potential in PBS solution using a Zetasizer NanoZS (Malvern, Orsay, France) at 25°C. Negative staining for transmission electron microscopy (TEM) analysis was done for PLA nanoparticles coated with different concentrations of RGDS-protein as previously described [27]. TEM observations were done with a Philips CM120 transmission electron microscope at the "Centre Technologique des Microstructures" (Lyon, France), and images were captured using a Gatan Orius 200 2Kx2K Camera. Particle size distribution was determined using Image J software.

### PLA nanoparticles coated with recombinant proteins

For the formulation assays, equal volumes of PLA nanoparticles (1%) and recombinant proteins in PBS were mixed, and the resultant solution was kept for 2 h at room temperature with moderate overhead stirring. Protein adsorption on the surface of these negatively charged particles is a combination of hydrophobic and electrostatic interactions. To make PLA nanoparticles coated with both p24 and RGDS- or KGES-FNIII-9/10 proteins, the same experimental procedure was followed except that proteins were added sequentially. Hence, PLA nanoparticles coated with p24 were mixed with one of the fibronectin fragments, and the solution again underwent a moderate overhead stirring for 2h at room temperature. To estimate the protein adsorption yield at the surface of nanoparticles, the solution was centrifuged at 8,000 x g for 10 min at 4°C, and the concentration in the supernatant of unbound proteins was determined using the Coomassie (Bradford) protein assay kit. Briefly, 150 μL of standard of bovine serum albumin (BSA) or working sample were mixed with 150 μL of Coomassie reagent, and the microplate was incubated 10 min at room temperature. The absorbance was measured at 595 nm on a plate reader. The working range was comprised between 1 and 25 μL. Thermodynamic diameter and zeta potential of all formulations were performed as indicated above.

### Cell adhesion and cell inhibition assays

Cell adhesion assays were carried out as previously described [23]. Briefly, different concentrations of recombinant proteins were coated in triplicate on Maxisorp 96 well plates, and after saturation with BSA, 40,000 HT-1080 cells were added to the wells and the microplates incubated at 37°C for 45 min. Non-adherent cells were removed and the remaining were fixed using the buoyancy method [28]. After staining cells with crystal violet, and cell lysis with 2% sodium dodecyl sulfate, cell adhesion to its substratum was quantified by measuring the optical density at 560 nm in a microplate reader (Multiskan FC; Thermo Fisher Scientific, Villebon sur Yvette, France). For inhibition assays, function-blocking anti-integrin antibodies were added to the cell suspension before addition in protein-coated wells.

### Flow cytometric analysis

For these experiment, 5X10^5^ cells were grown overnight at 37°C in a 5% CO_2_ atmosphere in Dulbecco's modified Eagle's medium (DMEM) supplemented with 10% fetal bovine serum. Cells were then washed twice with PBS, and Coumarin 6-PLA nanoparticles coated or not with fibronectin recombinant proteins diluted in PBS were added (20,000 nanoparticles/cell from a PLA/protein batch of 0.5% /10 μg/mL). After different times of contact, cells were washed twice in PBS, removed from the plates with 0.25% trypsin/0.05% EDTA, and fixed with 3% paraformaldehyde (PFA). Flow cytometric acquisitions were performed on a LSR II cytometer (BD Biosciences, New Jersey, USA) on the platform "AniRA cytométrie" of the SFR BioSciences (UMS3444/US8). Five thousand events per sample were acquired by FACS, and data analysis was done using the Flowjo 7 software (Tree Star, Ashland). This experiment was done twice without replicates for each assay.

### Fluorimetric analysis

HT-1080 cells resuspended in DMEM with 10% FBS (4X10^5^ cells/mL) were distributed in 96-well plates (100 μL per well), and incubated overnight at 37°C, 5% CO2. After washing twice with PBS, different concentrations of Coumarin 6-PLA nanoparticles coated or not with recombinant proteins and diluted in PBS, were added, and the plates incubated at 37°C 5% CO2. Cells were then washed twice in PBS, resuspended in 100 μL PBS, and fluorescence quantification performed on a TECAN Infinite^®^ M1000 pro, a multi-mode microplate reader (TECAN, Lyon, France). For integrin inhibition assays, a supplemental step was added before contact of the PLA nanoparticles with cells, and consisted of the addition of an anti-α5 blocking antibody (10 μg/mL). This experiment was done in triplicate.

### Confocal microscopy analysis

HT-1080 cells (10^5^/mL) were seeded on glass coverslips and incubated overnight at 37°C 5% CO_2_. Then, BODIPY 500/510-PLA nanoparticles (100 μg/mL) coated with RGDS- or KGES-FNIII9/10 recombinant proteins were added and the cells incubated 15 min at 37°C, 5% CO_2_. To stain the cell membranes, CellMask™ deep Red plasma membrane Stain (5 μg/mL, Life Technologies) was added for the last 5 min. After three washing steps with PBS, cells were fixed with 3.7% paraformaldehyde for 20 min. After three further washing steps, z-stacks from cells were acquired using a LSM 710 confocal microscope ("Centre Technologique des Microstructures" (Lyon, France)), and the data collected analyzed using the Macro Particle_in cell_3D file from ImageJ software. With this program it was possible to distinguish between the intracellular and membrane regions, and to estimate endocytosed and membrane-bound nanoparticles [29]. This experiment was carried out once.

### Mice vaccination assays

Twenty-eight 6-week-old female BALB/c mice were purchased from Charles River laboratories (Ecully, France). This experiment was done at the "Plateau de Biologie Expérimentale de la Souris, Ecole Normale Supérieure de Lyon, France), following approval by the local ethics committee for animal experimentation (CECCAPP: Comité d'Evaluation Commun au Centre Léon Bérard, à l'Animalerie de transit de l'ENS, au PBES et au laboratoire P4; CECCAPP _ENS_2014_040), and according to institutional guidelines. Euthanasia of mice was carried out by CO_2_ exposures. Mice were subcutaneously immunized by injection in the right hind upper leg on days 0, 14 and 28, and sera, vaginal lavages and fecal pellets were collected one day before each injection, and on day 42. Sera, vaginal lavages and fecal pellets were obtained and treated as previously described [30]. Seven groups of 4 mice were determined for this experimental vaccination. For three groups, the 100 μL injections corresponded to PLA nanoparticles (0.25%) coated with RGDS- or KGES-FNIII-9/10 proteins (10 μg/mL) or p24 (100 μg/mL). The two vaccine prospective groups corresponded to 5 mice per group and these were injected with PLA nanoparticles (0.25%) that were co-coated with p24 (100 μg/mL) and RGDS- or KGES-FNIII-9/10 molecules (10 μg/mL). Finally, to evaluate the immunogenicity of the fibronectin recombinant fragments alone, the two last two groups were immunized with RGDS or KGES proteins (10 μg/mL) and 0.6% of a vaccine Alhydrogel^®^ adjuvant corresponding to an aluminium hydroxide wet gel suspension (Invivogen, Toulouse, France). Enzyme-linked immunosorbent assays (ELISA) were performed to evaluate fibronectin and p24 specifics IgA, IgM and IgG, on days 0, 13, 27, and 42 in sera, vaginal lavages and fecal preparations. Recombinant proteins p24, RGDS- or KGES-FNIII-9/10 recombinant proteins (100 μL of 1 μg/mL) were coated overnight at 4°C on 96-well Nunc maxisorp plates. After protein solution removal, wells were saturated for non-specific binding with 10% non-fat dried milk diluted in PBS for 1 h at 37°C. The plates were again washed three times with PBS/0.05% Tween-20. Serial dilutions in Dulbeco's PBS (DPBS)/1% BSA of sera, vaginal lavages and feces samples were added into wells (duplicate), and the plates were incubated 1 h at 37°C. After a further washing step, anti-mouse IgA, IgG or IgM coupled to horseradish peroxidase (HRP) at a 1:10,000 dilution (from Southern Biotech, Nanterre, France; Reference numbers, 1030–05, 1040–05 and 1020–05) was added and the plates were incubated 1 h at 37°C. Plates were washed three times with PBS/0.05% Tween-20, and HRP reaction were done by adding 100 μL of tetramethylbenzidine substrate (BD biosciences, Paris, France) per well. After the addition of 1N H_2_SO_4_ to stop the reaction, the optical densities (OD) at 450 nm and 630 nm were measured in a microplate reader. To estimate the avidity of the immunoglobulins, we have tested antibody/antigen binding resistance to 8 M urea using a published method [31]. A prerequisite step was to have serum dilutions giving an OD at 450 nm of between 1 and 1.5. The protein p24 (100 μL of 1 μg/mL) was coated overnight at 4°C on 96-well Nunc maxisorp plates. After protein solution removal, wells were saturated for non-specific binding with 10% non-fat dried milk diluted in PBS for 1 h at 37°C. After three washing steps with PBS/0.05% Tween-20, serum samples diluted in DPBS/0.05% Tween-20 were added to the wells (duplicate), and the plates were incubated 1 h at 37°C. The plates were then washed three times with PBS/0.05% Tween-20 or PBS/0.05% Tween-20/urea 8 M. The last steps were performed as for the ELISA assays. According to Mann et al. [31], the avidity index corresponds to OD450 of urea-treated samples/OD450 of PBS/0.05% Tween-20 samples.

### Statistical analyses

Each result shown is from an experiment representative of at least three independently repeated experiments. Statistical analysis of the different assays was performed using the two-tailed paired Student’s t test. A value of P < 0.05 was considered statistically significant (asterisks in figures).

## Results

**Fig 1:**
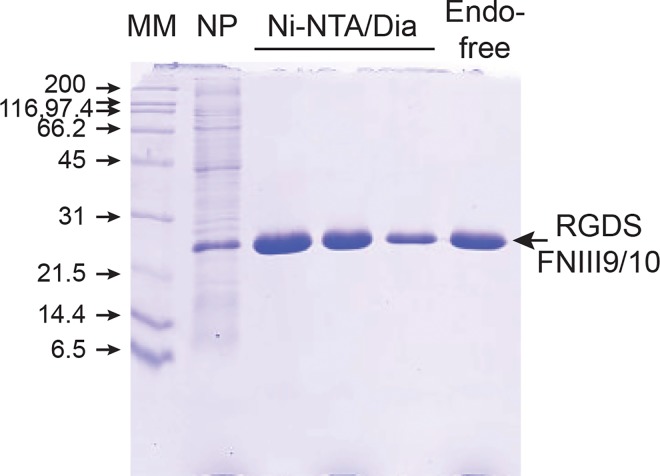
Purification of endotoxin-free RGDS-FNIII9/10 recombinant protein. After production in BL21(DE3) (NP, unpurified soluble protein fraction), His-tagged proteins were purified on a Ni-NTA chromatography column and dialyzed (Dia) against 20 mM phosphate buffer pH7. The last step was the removal of endotoxin (Endo) from the recombinant protein stock. Protein fractions were analyzed by SDS-PAGE (15%). Comparable data were obtained for KGES-FNIII9/10 recombinant protein (data not shown). MM, Molecular mass markers.

Wild-type recombinant protein used in this study consisted of FNIII domains 9 and 10 of the human fibronectin that are involved in RGD-dependent binding to α5β1 integrin. The tripeptide RGD sequence is located in the FNIII-10 domain and its interaction with the α5β1 integrin strongly reinforced by the synergy sequence PHSRN present in the FNIII-9 repeat [32–34]. C-terminal His-tagged RGDS-FNIII9/10 and the mutant KGES-FNIII9/10 recombinant proteins were overexpressed in soluble form in bacteria and purified to homogeneity on a Nickel-agarose affinity column. For their further use as bioreagents, the potent pro-inflammatory endotoxins were removed until an acceptable level was reached for medical use (<5 EU/mL; 1.9 to 2.5 EU/ml for the protein stocks used in this study) (Fig 1). To test the functional integrity of these recombinant proteins, cell adhesion and inhibition assays were performed. As shown in S1A Fig, α5β1-expressing HT-1080 cells [35] adhered to RGDS-FNIII9/10 but not to KGES-FNIII9/10 coated plates. The RGD-mediated interaction was significantly reduced when HT-1080 cells were preincubated with anti-α5 or anti-β1 integrin blocking antibodies before the cell-adhesion assays (S1B Fig). These results are comparable to previous studies, and confirm the structural integrity of the integrin-binding domain [34, 36–37].

**Fig 2:**
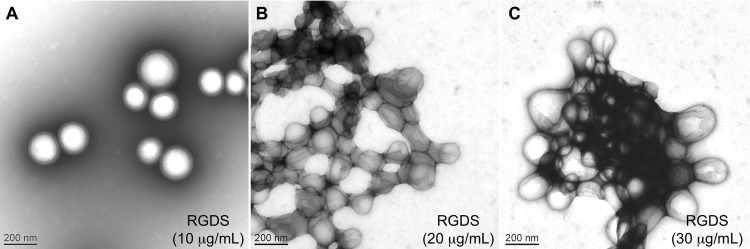
Transmission electron microscopy images of PLA-nanoparticles coated with RGDS-FNIII-9/10. Images were made after 2 h of incubation at room temperature in a PBS solution of PLA nanoparticles (0.5%) with 10 μg/mL (A), 20 μg/mL (B) and 30 μg/mL (C) of RGDS-FNIII-9/10 recombinant protein.

With the aim to develop a nanocarrier vaccine delivery system, we first carried out a dose-formulation pilot study of PLA nanoparticle-recombinant protein interactions in PBS solution. Hydrodynamic diameters and the corresponding polydispersity index (PDI), plus percentages of proteins coated in the different nanoparticle formulations are presented in Table 1. Blank PLA nanoparticles had a hydrodynamic diameter of 193 nm and a PDI of 0.059 characteristic of a monodispersed biomaterial size distribution. DLS analyses indicated a slight increase in the hydrodynamic diameter of PLA nanoparticles after 2 hours of contact with 10 μg/mL of RGDS- (207 nm) or KGES-FNIII9/10 (206 nm) proteins while their PDI values were not affected. Increasing by two or three times the protein concentration led to an increase in the PLA nanoparticle hydrodynamic diameter. Comparable results were observed for the PDI, with the polydispersity increasing with the protein concentrations. Protein adsorption at the surface of the PLA nanoparticles was efficient, almost 100% for most of the experiments (Table 1). It should be noted that for protein concentrations higher than 20 μg/mL, a large heterogeneity in the results was obtained (hydrodynamic diameter, PDI), regardless of the recombinant peptide used. To better appreciate the status (form, monodispersity, agglomeration and aggregation) of the decorated PLA nanoparticles, TEM analysis was performed using the RGDS-FNIII9/10 nanomaterial (Fig 2). At the lowest RGDS-FNIII9/10 protein concentration (10 μg/mL), the nanoparticles were spherical in shape and presented a unimodal size distribution with an average diameter of 200 nm ± 45 nm. At twice the concentration (20 μg/mL), the PLA nanoparticles appeared agglomerated and at the highest concentration, large particle aggregates were observed.

**Table 1:** Characterization of PLA nanoparticles in PBS solution.

PLA (0.5%)	blank	RGDS-FNIII9/10			KGES-FNIII9/10		
μg/mL protein	0	10	20	30	10	20	30
hydrodynamic diameter (nm)	193±4	207±12	238±14	340±17	206±13	223±12	241±13
Polydispersity Index	0.059±0.01	0.089±0.02	0.127±0.02	0.328±0.01	0.052±0.004	0.091±0.01	0.122±0.02
% coated protein*	-	98.6	96	87.1	100	95.3	91.4

(*) Low levels of non-adsorbed protein molecules might be underestimated due to the working range (1–25 μg/mL) of the Coomassie protein assay.

**Fig 3:**
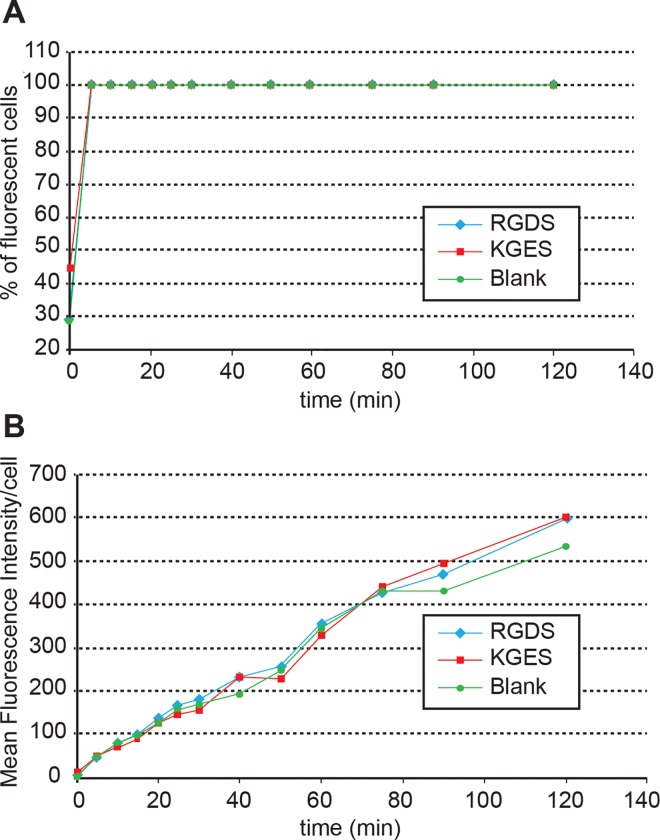
Time-scale analysis of the cellular uptake of PLA nanoparticles coated or not, with recombinant proteins. After different times of contact between Coumarin 6-PLA nanoparticle/protein batches (0.5%/ 10 μg/mL) and HT-1080 cells (20,000 nanoparticles/cell), the percentage of fluorescent cells (A) and the mean fluorescence intensity/cell (B) were monitored by flow cytometry.

**Fig 4:**
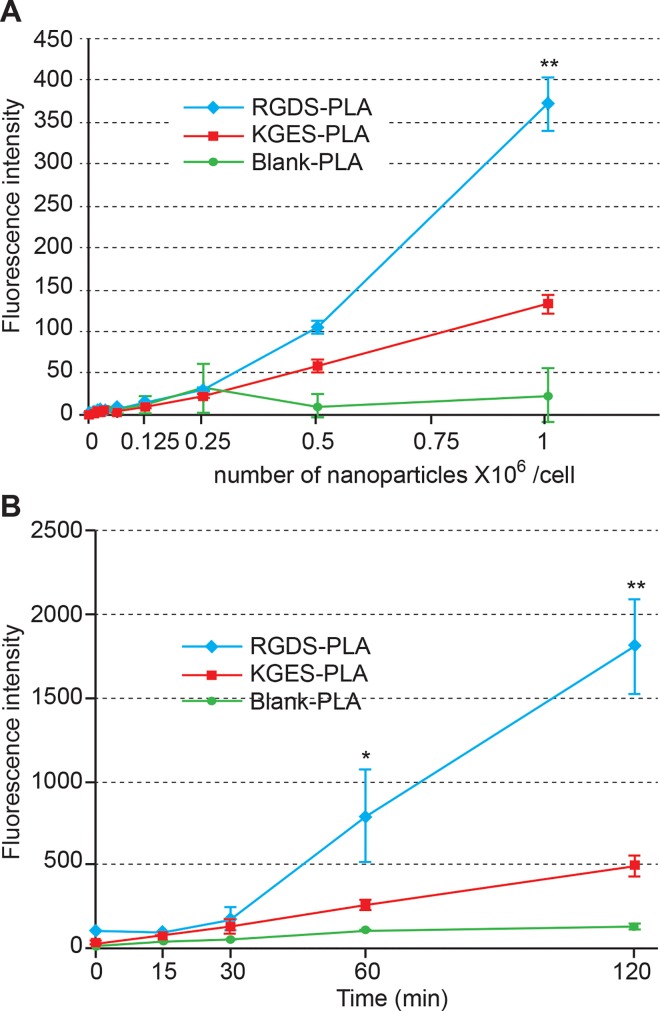
Fluorimetric analysis of the cellular uptake of PLA-nanoparticles. (A) Dose-dependent cellular uptake of PLA nanoparticles after 90 min of incubation at 37°C. (B) Time-scale of cellular uptake of PLA nanoparticles (10^6^ nanoparticles/cell). Fluorescence intensity was measured on a multi-mode microplate TECAN Infinite^®^ M1000 pro reader. Bars represent the S.D. of triplicate assays. Significant (*, P<0.05; **, P<0.005) difference versus vehicle assay.

**Fig 5:**
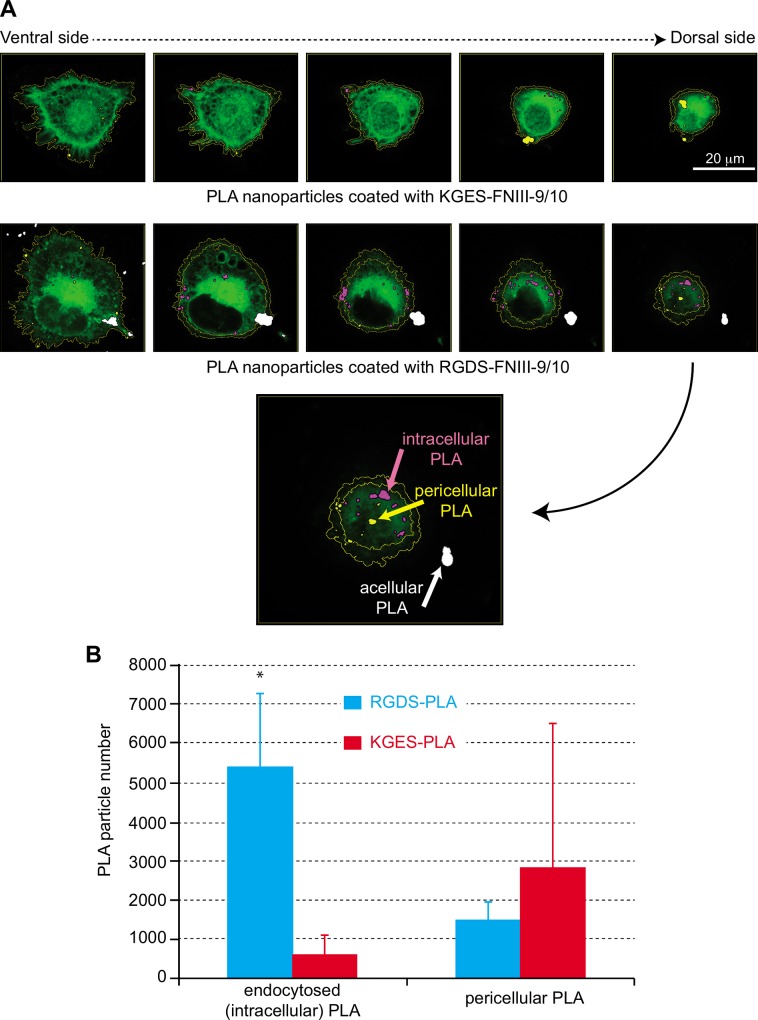
Confocal microscopy analysis of cellular uptake of PLA-nanoparticles. (A) Z-stack images of cells incubated with BODIPY PLA nanoparticles coated with KGES- or RGDS-FNIII-9/10 recombinant proteins. Intracellular, pericellular and acellular PLA-nanoparticles are stained in pink, yellow and white, respectively. The green staining represents the CellTracker™ Red CMTPX. (B) Distribution of intracellular and pericellular PLA nanoparticles determined from Z-stack analysis. Significant (*, P<0.05) difference versus KGES assay.

To analyze the influence of the recombinant proteins on nanoparticle cell-uptake, we chose the concentration (10 μg/mL) of proteins for which no aggregates were observed by TEM analysis. The PBS culture condition was chosen to avoid any interference with fibronectin fragments present in fetal bovine serum. For the following experiments, uncoated or RGDS- or KGES-FNIII9/10 coated PLA nanoparticle batches containing vital-dye (Coumarin 6) were used. As a first step, over two hours we evaluated the interaction and kinetics of internalization of the different sets of Coumarin 6-PLA nanoparticles on HT-1080 cells. Flow cytometric data shown in Fig 3 indicate that all cells were stained 6 min after the deposition of the PLA nanoparticles in the cell culture (Fig 3A), and that the amount of fluorescence/cell increased progressively during this period throughout the different assays (Fig 3B). In a second step and to avoid the trypsinization used for flow cytometric analysis, cell staining intensity was determined using a TECAN multi-well reader. In this experiment, different concentrations of PLA-nanoparticles batches were added to culture assays, and the cells were incubated for 90 min. As shown in Fig 4A, RGDS-FNIII9/10 coated PLA-nanoparticles bound more or were more endocytosed by HT-1080 than KGES-FNIII9/10 particles (twice) and blank nanocarriers (8 times), but the difference was only highly significant (P<0.005) at the highest concentration (1 million PLA nanoparticles/cell). A time course study carried out at this concentration confirmed a significant difference in the integrin-binding coated PLA nanoparticles versus the others assays, after 60 min (Fig 4B). The specificity of the RGDS-FNIII9/10 coated PLA-nanoparticles for integrin α5β1 was tested using anti-α5β1 or anti α203B21 blocking antibodies (S2 Fig). A significant (P<0.005) decrease in fluorescence intensity (4 times) for HT-1080 cells was obtained using anti-α5 antibody versus vehicle sample, while the uptake of RGDS-FNIII9/10 coated PLA-nanoparticles was even greater in the presence of anti-α2 blocking antibody. To analyze more precisely whether the difference in cell staining intensity observed between RGDS and KGES nanoparticle experiments was due to an increase in binding to the plasma membrane or to a more efficient endocytosis of the nanocarriers, a confocal microscopy analysis was carried out on HT-1080 cells after 15 min of contact with BODIPY-PLA nanoparticles. A series of z-stack confocal microscopy images of cells (from the ventral or attached side to the dorsal side) in contact with RGDS- or KGES-FNIII-9/10 PLA nanoparticles is shown in Fig 5A, and the estimated number of nanoparticles either at the surface or inside the cells is presented in Fig 5B. A significant increase in cellular endocytosis was observed with α5β1-binding RGDS- versus KGES-coated PLA nanoparticles.

**Fig 6:**
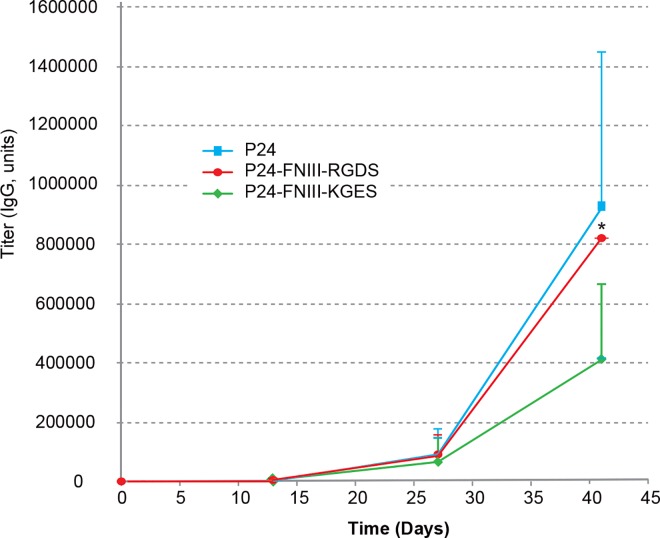
p24 specific IgG responses in mouse sera after injection of PLA nanoparticles coated with p24 (100 μg/mL) or co-coated with p24 (100 μg/mL) and RGDS or KGES recombinant proteins (10 μg/mL). Data represent the p24 specific IgG titer at D0, D13, D27 and D42. The IgG values represent the average of 4 (p24) or 5 (p24 + RGDS or KGES) mouse assays. Error bars are means + SD. Significant (*, P<0.05) difference versus KGES assay.

In order to test the potential and the interest of RGDS nanoparticles *in vivo*, we carried out a vaccination approach in a mouse model, by using p24, an HIV capsid protein as antigen, and its co-coating with RGDS- or KGES-FNIII-9/10 to PLA nanoparticles. The characteristics of the different sets of PLA nanoparticles (hydrodynamic diameter, PDI, zeta potential, percentage of protein coating) are shown in S1 Table. The hydrodynamic diameter of PLA nanoparticles coated with p24 was slightly larger (223 nm instead of 210 nm for the blank particles). A still acceptable PDI (< 0.1), and an increase in the hydrodynamic diameter were observed after the addition of RGDS (237 nm) or KGES (238 nm) recombinant proteins. The immunogenic potential of the different formulations were determined after subcutaneous injections in BALB/c female mice, and anti-p24 and anti-FNIII-9/10 antibodies (IgA, IgG and IgM) were titrated from sera, vaginal lavage fluids and feces on days 0, 13, 27, and 41. As shown in Fig 6, there was no significant difference in the anti-p24 IgG serum titer in mice injected with PLA nanoparticles coated with p24, alone or in the presence of RGDS- or KGES-FNIII-9/10, although the integrin-binding recombinant protein was significantly more efficient (IgG titer) than its mutated counterpart. No immunostimulatory switch towards mucosal immunity was determined for seric IgA, or for vaginal and fecal IgG or IgA. Titers of anti-human fibronectin in PLA-injected mice were not negligible (S3 Fig), but they were 10-fold lower than those obtained for p24.

## Discussion

In this study, we developed a biomaterial vehicle to target more efficiently cells presenting the α5β1 integrin at their surface. Hence, in cell culture assays, the presence of the RGDS sequence on the surface of PLA-nanoparticles mediated a stronger uptake of these nanocarriers as judged by fluorimetric and confocal microscopy analyses. We observed no apparent advantage to using wild-type or mutated fibronectin recombinant proteins to improve the humoral response potential of an antigen coating on the surface of these PLA nanoparticles after subcutaneous injections in mice. However, the presence of fibronectin proteins on the surface of p24-PLA nanoparticles increased the quality of the humoral immune response, as judged by the avidity index analysis.

Adhesion and anti-integrin inhibition assays confirmed that the recombinant proteins expressed in *E*. *coli* maintained their structural properties, allowing their binding to α5β1 integrin. However, nanoparticle aggregation was observed at the highest recombinant protein concentrations used, and might correspond to the interaction between FNIII molecules and two or less PLA particles, probably due to lateral domain interaction between two FNIII9/10 molecules. It should be noted that the interaction of FNIII1/2 with the N-terminal domain of fibronectin has been suggested to be an important factor in fibronectin fibrillogenesis [38]. Interaction between fibronectin FNIII2/3 and FNIII12-14 domains has also been demonstrated [39].

Several experiments were done to test the potential of wild type or mutated fibronectin fragments to favor α5β1 integrin cellular-uptake after association with PLA nanoparticles. Based on other studies [40–42], a first approach to analyze the cellular uptake of the different sets of nanoparticles was to use flow cytometry to monitor the fluorescence intensity of cells. Cellular-uptake of nanoparticles was a fast process in our experimental procedure (Fig 3A). However, the presence of RGDS-FNIII9/10 on the surface of the nanoparticles did not seem to increase the efficiency of their uptake by α5β1 HT-1080 cells (Fig 3B), in comparison to vehicle or KGES-FNIII9/10 assays. Apart from a structural modification of the RGDS protein on the surface of these nanoparticles, several factors might interfere with the flow cytometry experiment. It has been shown that, in cell culture assays, the percentage of nanoparticles in contact with cells is less than 0.33% [43]. Moreover, the trypsin treatment of cells before their flow cytometric analysis might have perturbed the plasma membrane and influenced their cellular uptake. Another limitation of this method is that we cannot differentiate nanoparticles that are within the cells to those present on their surface.

We therefore used a fluorimetric method that did not require a trypsin step, and used a broad concentration of PLA nanoparticles to better analyze the impact of the surface-coated RGDS-FNIII9/10 recombinant protein on the efficiency of cellular uptake. The data confirm that, for concentrations higher than 500,000 nanoparticles/cell, the presence of the RGDS sequence favors the cellular uptake of nanoparticles. However, the exact location of the nanoparticles (across the plasma membrane or inside the cell) could not been resolved, although the presence of anti α5 antibodies drastically inhibited cellular uptake (S2 Fig) and suggests that this fibronectin fragment maintains its functionality at the surface of the nanoparticles. The use of confocal microscopy permitted us to confirm that nanoparticles decorated with RGDS protein are more efficiently taken up by HT-1080 cells than uncoated or KGES PLA nanoparticles.

**Fig 7:**
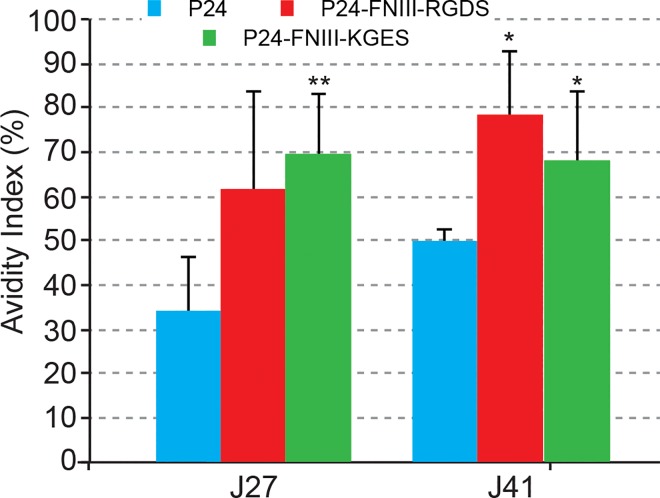
Anti-p24 antibody avidity index. Serum samples of mice after 27 and 41 days of immunization with p24, RGDS/p24 or KGES/p24 PLA nanoparticles were used to estimate the avidity index as a percentage. The average index corresponded to the OD_450_ of urea-treated samples divided by OD_450_ of PBS/0.05% Tween-20 samples. Significant (*, P<0.05; **, P<0.005) difference versus p24 assay.

Our attempt to administer RGDS nanomaterial as a subcutaneous vaccine adjuvant and delivery vehicle did not improve the production of anti-p24 antigen antibodies, but as this was a pilot experiment, we did not investigate the cellular immune response, and we could not exclude the possibility that this response had been improved, as cross-presentation could have been enhanced, through a better uptake of FNIII-PLA nanoparticles by antigen-presenting cells. The quality of the humoral immune response presented in Fig 7 suggested that the presence of such fibronectin FNIII domains favored the migration of PLA nanoparticles to the lymph nodes, therefore enhancing the quality of antibodies, in terms of avidity. Additional experiments are ongoing to explore such a hypothesis. An additional point, which deserves attention, concerns the induction of anti-FNIII antibodies (S3 Fig). This could provoke the induction of auto-antibodies that would be detrimental to the use of these integrin targeting approaches for prophylactic vaccines. However, the induction of such antibodies could be due to the 15% divergence between mouse and human sequences.

After 41 days of vaccination, the serum avidity index of mice treated with RGDS/p24 nanoparticles seemed to be higher, but not statistically significant, than that corresponding to KGES/p24 samples. We can hypothesize that this apparent loss of function of the RGDS sequence *in vivo*, may be related to different aspects of the experimental procedures, through the choice of immunization route or the number of mice used in this study. Indeed, a dermal injection procedure instead of the subcutaneous approach used in this study may favor the targeting of dendritic cells as they will be denser when reached through such a route, and favor a better cellular immune response [44–45]. In line with this observation, we also propose to test other delivery procedures, such as mucosal administration. Lastly, we cannot exclude, at the biochemical level, that the co-coating of fibronectin fragments with p24 antigen could also interfere with the binding of the RGDS sequence with related integrins, perhaps by sterically preventing the RGDS-integrin interaction. It should be noted that we used 10 times less of fibronectin recombinant protein than the p24 antigen (10 μg/mL versus 100 μg/mL). New vaccine approaches with different fibronectin/p24 protein ratios could potentially improve the use of the RGDS sequence. More experiments need to be performed to develop such new innovative targeted vaccines, but cell culture experiments and mouse immunization results clearly suggest their future potential.

## Conclusion

In this study, we have developed PLA nanoparticles capable of targeting cells presenting α5β1 integrins at their surface, by adsorbing RGDS-FNIII repeats from fibronectin on their surface. Hence, the presence of the RGDS sequence facilitates nanoparticle uptake by α5β1-positive cells, as demonstrated by confocal microscopy analysis. Subcutaneous injections of mice with PLA nanoparticles co-coated with an antigen (p24 HIV capsid protein, here) and RGDS-recombinant proteins did not elevate the anti-24 IgG titer in the serum in comparison to a vaccine made of p24-PLA nanoparticles, but the avidity index of the antibodies was significantly increased.

## Supporting Information

S1 Fig HT-1080 cell adhesion to RGDS- and KGES-FNIII-9/10 recombinant proteins. (A) Dose-dependent adhesion of HT-1080 cells to recombinant proteins. Wells from microplates were coated with increasing concentrations of RGDS- or KGES-FNIII-9/10 recombinant proteins, and cell adhesion was monitored after 45 min of contact with "adhesive" surface at 37°C and crystal violet staining. (B) Inhibition of HT-1080 cell adhesion to RGDS-FNIII-9/10 recombinant protein in the presence of anti-integrin antibodies. HT-1080 cells were incubated with or without antibodies before plating in wells coated with RGDS-FNIII-9/10 (20 μg/mL). Bars represent S.D. of triplicate assays. Significant (**, P<0.005) difference versus control assay without anti-integrin antibodies is indicated.
(EPS)

S2 Fig Inhibition assays of PLA nanoparticles endocytosis. The experimental procedure was performed as in Fig 4, except that anti-integrin α2 or α5 was added to the cells before the addition of PLA nanoparticles coated with RGDS-FNIII-9/10. Fluorescence intensity was measured on a multi-mode microplate TECAN Infinite^®^ M1000 pro reader. Bars represent the S.D. of triplicate assays. Significant (**, P<0.005) difference versus control assay.
(EPS)

S3 Fig KGES-FNIII9/10 specific IgG responses in mouse sera after injection of PLA nanoparticles coated with RGDS or KGES recombinant proteins (10 μg/mL). Data represent the KGES-FNIII9/10 specific IgG titer on D0, D13, D27 and D42. The IgG values represent the average of 4 mouse assays. Error bars are means + SD.
(EPS)

S1 Table Characteristics of PLA nanoparticle batches used for subcutaneous injections (a new set of nanoparticle solutions was prepared before each injection). (DOCX)
