# Supplementary material for: Poly(Lactic Acid) Nanoparticles Targeting α5β1 Integrin as Vaccine Delivery Vehicle, a Prospective Study
Source: PLoS One. 2016 Dec 14;11(12):e0167663. doi: 10.1371/journal.pone.0167663 (PMC5156357; doi:10.1371/journal.pone.0167663)
Supplement: S1 Table — (DOCX) [file pone.0167663.s004.docx]

**S1 Table. Characteristics of PLA nanoparticle batches used for subcutaneous injections (a new set of nanoparticle solutions was prepared before each injection)**

|  | Hydrodynamic diameter (nm) | Polydispersity  Index | Zeta potential  (mV) |
| --- | --- | --- | --- |
| PLA (1%) | 210±1 | 0.017±0.01 | -63.95±1.29 |
| PLA nanoparticles (0.5%) + p24 (200 μg/mL) | 223±5 | 0.039±0.02 | -56.6±0.83 |
| PLA nanoparticles (0.25%) +  p24 (100 μg/mL)  + RGDS (10 μg/mL) | 237±5 | 0.079±0.01 | -54.08±1.14 |
| PLA nanoparticles (0.25%) +  p24 (100 μg/mL)  + KGES (10 μg/mL) | 238±15 | 0.07±0.01 | -48.53±0.95 |
